# Analyzing Sex-Specific Dimorphism in Human Skeletal Stem Cells

**DOI:** 10.3390/cells12232683

**Published:** 2023-11-22

**Authors:** Tarek Niemann, Jonas Joneleit, Jonathan Storm, Tom Nacke, Dirk Wähnert, Christian Kaltschmidt, Thomas Vordemvenne, Barbara Kaltschmidt

**Affiliations:** 1 Molecular Neurobiology, Bielefeld University, Universitätsstrasse 25, 33615 Bielefeld, Germany; jonas.joneleit@uni-bielefeld.de (J.J.); tom.nacke@uni-bielefeld.de (T.N.); barbara.kaltschmidt@uni-bielefeld.de (B.K.); 2 Forschungsverbund BioMedizin Bielefeld FBMB e.V., 33615 Bielefeld, Germany; jonathan.storm@uni-bielefeld.de (J.S.); dirk.waehnert@evkb.de (D.W.); c.kaltschmidt@uni-bielefeld.de (C.K.); thomas.vordemvenne@evkb.de (T.V.); 3 Department of Cell Biology, Bielefeld University, Universitätsstrasse 25, 33615 Bielefeld, Germany; 4 Department of Trauma and Orthopedic Surgery, Protestant Hospital of Bethel Foundation, Campus Bielefeld-Bethel, University Hospital OWL of Bielefeld University, Burgsteig 13, 33617 Bielefeld, Germany

**Keywords:** skeletal stem cells, sex dimorphism, osteogenic differentiation, inflammation, proliferation, NF-κB

## Abstract

Sex-related differences are a current topic in contemporary science. In addition to hormonal regulation, cell-autonomous mechanisms are important in bone homeostasis and regeneration. In this study, human skeletal stem cells (SSCs) from female and male adults were cultured and analyzed with immunological assays and osteogenic differentiation assessments. Female SSCs exhibited a mean doubling time of 100.6 h, whereas male SSCs displayed a mean doubling time of 168.0 h. Immunophenotyping revealed the expression of the stem cell markers Nestin, CD133, and CD164, accompanied by the neural-crest marker SOX9. Furthermore, multiparameter flow cytometric analyses revealed a substantial population of multipotent SSCs, comprising up to 80% in both sexes. An analysis of the osteogenic differentiation potential demonstrated a strong mineralization in both male and female SSCs under physiological conditions. Recognizing the prevailing association of bone diseases with inflammatory processes, we also analyzed the osteogenic potential of SSCs from both sexes under pro-inflammatory conditions. Upon TNF-α and IL-1β treatment, we observed no sexual dimorphism on osteogenesis. In summary, we demonstrated the successful isolation and characterization of SSCs capable of rapid osteogenic differentiation. Taken together, in vitro cultured SSCs might be a suitable model to study sexual dimorphisms and develop drugs for degenerative bone diseases.

## 1. Introduction

The skeletal system, consisting of different tissues that include bone and cartilage, is the human framework that provides structure, movement, and protection and is constantly stressed by mechanical effects. To maintain functional bone homeostasis, remodeling and regeneration processes must occur throughout life [1,2]. These processes are driven by newly discovered human skeletal stem cells (SSCs), which have been identified as playing a role in the growth, maintenance, and regeneration of the bony structure [3]. While the characterization of the SSCs is becoming more advanced, the exact functions of SSCs in regenerative processes remain elusive. SSCs are mainly enriched in the hypertrophic growth plate of the bones [4] but can also occur in the perichondrium and the periosteum [5]. In recent years, different subpopulations of skeletal stem cells have been identified using fluorescence activated cell sorting (FACS) along with lineage tracing [6,7]. These findings led to the definition of SSCs as cells with the ability to perform self-renewal; and the capability of multilineage differentiation to bone, cartilage, and stroma; as well as cells that express specific immunophenotypic markers (PDPN^+^CD146^−^CD73^+^CD164^+^) [8]. The differentiation of skeletal stem cells via bone, cartilage, and stromal progenitors (BCSPs, PDPN^+^CD146^+^) can lead to the formation of two distinct subsets of osteoprogenitors (OPs, PDPN^−^CD146^+^CD90^high^, PDPN^−^CD146^+^CD90^low^) and three subsets of chondrogenic progenitors (CPs, PDPN^+^CD146^−^CD73^−^CD164^−^, PDPN^+^CD146^−^CD73^+^CD164^−^, PDPN^+^CD146^−^CD73^−^CD164^−^) [9]. Taken together, at least two types of multipotent SSCs exist. According to their predominance in specific regions, they can be termed epiphyseal SSCs (eSSCs, CD164^+^, PDPN^+^, CD73^+^, Lin^−^, CD45^−^, CD146^−^) and perivascular SSCs (pSSCs, PDPN^+^, CD146^+^).

Notably, SSCs are capable of building multilineage ossicles in vivo [8]. Understanding the role of SSCs in the remodeling processes could lead to improved development of cellular therapies for degenerative bone diseases.

The vulnerability to bone disorders and variations in bone composition and density between the sexes are widely investigated [10,11,12]. Particularly, osteoporosis affects women more frequently than men with a prevalence of 23.1% in women compared to 11.7% in men [13]. Likewise, having lower bone density is considered a major risk factor for developing fractures in women [14,15]. Bone homeostasis is strongly influenced by hormones and stem cell behavior [16,17]. Estrogens can inhibit osteoclast formation, enhance bone mineralization, and promote osteoblast survival [18]. Declining estrogen levels due to menopause is the primary cause of bone loss and osteoporosis in women aged 50 years and older [19,20]. According to a murine study by Andrew and coworkers, skeletal stem cell-mediated bone regeneration in females is driven by estrogen signaling, while the male counterpart does not respond to estrogen [21]. Also, the differentiation and regeneration capacity of stem cells, which is crucial for regenerative and developmental processes, is highly influenced by sex [22,23]. A study by Greiner and colleagues has shown that female neural crest-derived stem cells have a reduced quantity of osteogenic stem cell compartments, leading to a reduced osteogenic differentiation potential [24]. Localized and systemic inflammatory responses are also involved in the development of osteoporosis, especially in women during menopause [25]. In particular, the proinflammatory cytokines tumor necrosis factor α (TNF-α) and interleukin 1β (IL-1β) suppress bone formation and enhance bone resorption by inhibiting osteoclast apoptosis and promoting osteoclast precursor proliferation [26]. Rheumatoid arthritis (RA), a bone disorder that affects both bones and joints, links osteoporosis to bone loss and inflammation enhanced by increased levels of TNF-α, IL-1β, and metalloproteinases [27,28]. Both cytokines TNF-α and IL-1β activate diverse signaling pathways that have the potential to enhance the inflammatory process by activating the nuclear factor ‘kappa-light-chain-enhancer’ of activated B cell (NF-κB) signaling [29].

In this study, we initially examined sex-specific differences in human SSCs by comparing their doubling time and cell size. Subsequently, we analyzed the cell populations with flow cytometry and performed immunocytochemistry to assess several stemness and neural crest-derived markers. In the following experiments, we analyzed potential sex-specific differences in the osteogenic differentiation potential of the SSCs under physiological conditions. Since inflammatory processes are involved in the development of osteoporosis and rheumatoid arthritis, which is more common in women, our study aimed to analyze potential sexual dimorphisms in the influence of an inflammatory environment on the osteogenic differentiation potential of SSCs. First, we stimulated SSCs of both sexes with the proinflammatory cytokines TNF-α and IL-1β and demonstrated nuclear translocation of NF-κB. We further evaluated the osteogenic differentiation potential of SSCs under these conditions, especially in the light of potential sex-specific differences during bone regeneration.

## 2. Materials and Methods

### 2.1. Isolation of Human Skeletal Stem Cells

Human skeletal stem cells were isolated from human femoral heads obtained during arthroplasty surgery performed by the department of trauma surgery and orthopedics of the protestantic Hospital of Bethel Foundation. All experimental procedures were ethically approved by the ethics board of the medical faculty of the University of Münster (No. 2012-015-f-S). SSCs were isolated using mechanical disintegration and enzymatic digestion, followed by density gradient centrifugation as depicted in Figure S1. The cell suspension was seeded into a T25 cell culture flask containing culture medium that consisted of DMEM/F12 (with phenol red) (PAN Biotech, Aidenbach, Germany), 1% penicillin/streptomycin (Sigma Aldrich, St. Louis, MO, USA), 2 mM L-glutamine (Sigma Aldrich, St. Louis, MO, USA), and 2% human platelet lysate (HPL, STEMCELL Technologies, Vancouver, BC, Canada).

### 2.2. Cell Culture

All work with cells was performed in biological safety cabinets in strict compliance with safety procedures for sterile work. The experiments were performed using isolated SSCs from eight male and eight female donors. The health of the donors is clarified as part of the clinical routine. In case of suspicious potential infections, laboratory diagnosis was performed immediately after a detailed anamnesis. Here, only donors without infectious diseases were included. Table 1 illustrates the age, sex, and diagnosis of each donor along with their staging according to the Kellgren and Lawrence classification [30].

SSCs were seeded in a T25 cell culture flask (Sarstedt, Nürnbrecht, Germany) coated with 0.1% gelatin (lab made) to achieve cell adherence. SSCs were grown in culture medium supplemented with 2% HPL in a humidified incubator (CB150, Binder, Tuttlingen, Germany) adjusted to an atmosphere with 5% CO_2_ and 37 °C. Every three days, the cells were fed with fresh culture medium (HPL-medium). The cells were passaged using trypsin/EDTA (Sigma Aldrich, St. Louis, MO, USA). The culture medium was discarded, and the cells were washed using 1× PBS. A total of 2 mL of trypsin/EDTA was added to each flask and incubated for 2 min at 37 °C. The detaching process was stopped by adding an equal amount of culture medium. The cell suspension was transferred into a 15 mL Falcon tube and centrifuged at 300× *g* for 5 min. The resulting cell pellet was resuspended in fresh culture medium and used for further experiments.

### 2.3. Proliferation Assay

In order to determine the doubling time of the SSCs, a total of 5 × 10^3^ cells at passage 2–3 were evenly seeded into a cell culture flask that had been pre-coated with a 0.1% gelatin solution. This process was performed in triplicate to ensure the accuracy and reliability of the results. The cells were fed with their respective culture medium at intervals of three days until they achieved a significant level of confluency. Subsequently, the number of cells was calculated utilizing a Neubauer chamber. Ultimately, the doubling time of the donors was calculated according to Korzyńska and Zychowicz [31]. For comparing the SSCs’ doubling time with MSC doubling time, we used Lonza MSCs from a male donor at passage 4 and female MSCs at passage 4 derived from adipose tissue. The MSCs were cultivated in a medium consisting of DMEM high glucose (PAN-Biotech, Aidenbach, Germany), 1% penicillin/streptomycin, 2 mM L-glutamine, and 10% sterile filtered fetal bovine serum (FBS, Sigma Aldrich, St. Louis, MO, USA).

### 2.4. Immunocytochemistry

Human skeletal stem cells at passage 2 or 3 were seeded in a µ-Slide 8 Well (ibidi, Graefelfing, Germany), which had been coated with 0.1% gelatin, with a density of 5 × 10^3^ cells. Immunocytochemistry was performed as previously described by Ruiz-Perera et al. [32]. Primary antibodies against p65 (rabbit, 1:400, Cell Signaling Technology, Danvers, MA, USA, mAb#8242), Nestin (mouse,1:200, Merck Millipore, Burlington, MA, USA, MAB353), CD164 (mouse, 1:100, Santa Cruz Biotechnology, Dallas, TX, USA, sc-271179), CD133 (rabbit, 1:100, Novus Biologicals, Centennial, CO, USA, NB120-16518,), SLUG (rabbit, 1:400, Cell Signaling Technology, C1967), SOX9 (rabbit,1:400, Invitrogen, Waltham, MA, USA, #702016,), p75 (mouse, 1:100, Sigma Aldrich, N5408), and β-actin (rabbit, 1:200, Cell Signaling Technology, #4970) were diluted in 1× PBS and incubated for 1 h at room temperature. Secondary fluorochrome-conjugated antibodies (1:300; goat anti-rabbit Alexa Fluor 555, goat anti-mouse Alexa Fluor 488, Invitrogen) were applied and incubated for 1 h at room temperature, followed by nuclear staining with 4′,6-Diamidin-2-phenylindol (DAPI, 1:1000, Sigma Aldrich). The samples were analyzed with the confocal laser scanning microscope Zeiss LSM 900 (Imaging Core Facility University of Bielefeld). Lastly, fluorescence images were processed with ImageJ. For the semiquantitative analysis of marker expression, the autofluorescence of unstained cells was used as a threshold for claiming cells as positive or negative for the investigated marker. Immunocytochemistry performed with β-actin antibodies was used to calculate the cell area. First, the image to be analyzed was loaded into Fiji version 2.1.4. followed by the “Make Binary” feature to detect the outline of the cells. Next, the ROI Manager was opened, and the individual cells were selected with the “Wand” icon. This feature automatically recognizes the outlines of the cells. If insufficient outlining was observed, individual cells were outlined manually using the pen function. The outlined cells were then added to the ROI Manager. Next, a scalebar with a defined size of 100 µm was added. Then, a straight line was drawn along the automatic scalebar. “Analyze” was selected followed by the “Set scale to mm^2^” function. Then, the area calculation of the cells was performed in the ROI Manager.

### 2.5. Flow Cytometry

For flow cytometric characterization, the cells of the first passage were cultured and harvested as described previously. After the counting of living cells using trypan blue exclusion, the cells were resuspended in PBSE + 2% BCS (1× PBS + 2% EDTA + 2% bovine calf serum) at a concentration of 20,000 cells in 58 µL and aliquoted accordingly. A total of 10 µL Human Seroblock (Bio-Rad, Hercules, CA, USA, #BUF070B) and 10 µL Tandem Signal Enhancer, human (Miltenyi Biotec, Bergisch Gladbach, Germany, #130-099-887) were added, and the samples were incubated for 10 min at room temperature. Fluorochrome-conjugated antibodies (PDPN-APC (Thermo Fisher Scientific, Waltham, MA, USA, #17-9381-42); CD146-PE/Cy7 (BioLegend, San Diego, CA, USA, #342010); CD73-FITC (BioLegend, #344016); CD164-PE (BioLegend, #324808); CD45-VioGreen (Miltenyi Biotec, #130-110-776)) were added according to the manufacturer’s guidelines. After a 10 min incubation at room temperature in the dark, the cells were washed twice with PBSE + 2% BCS before resuspension in 200 µL PBSE + 2% BCS with 100 ng/mL DAPI (Sigma Aldrich, #D9542). The samples were placed in Small Volume Sample Tubes 1 mL (Sysmex, Kobe, Japan, #04-2010) and measured using a Beckman Coulter Gallios flow cytometer with the Kaluza for Gallios software (Beckman Coulter, Brea, CA, USA, version 1.0.14029.14028) using the forward scatter settings for large particle size, IsoFlow Sheath Fluid (Beckman Coulter, #8546859), and a flow rate of ~30 µL/min. Cytometer performance was controlled using Flow-Check Pro Fluorospheres (Beckman Coulter, #A69183), and PMT currents were set using Rainbow Fluorescent Particles, 1 peak (3.0–3.4 µm)—Mid Range Intensity (BioLegend, #422905). FITC, PE, and PE/Cy7 signals were measured on the 488 nm laser line in the filter sets FL1 (550 DCSP–525 BP), FL2 (595 DCSP–575 BP), and FL5 (755 LP). APC was measured in filter set FL6 (710 DCSP–660 BP) on the 638 nm laser line, and DAPI and VioGreen were measured on the 405 nm laser line in filter sets FL9 (480 DCSP–450 BP 50) and FL10 (550 BP 40). Compensation was performed using the BD™ CompBead Plus Anti-Mouse Ig, κ/Negative Control (BSA) Compensation Plus (7.5 µm) Particles Set (BD Biosciences, Franklin Lakes, NJ, USA, #560497) and the MACS^®^ Comp Bead Kit, anti-REA (Miltenyi Biotec, #130-104-693) for the antibodies and cells for DAPI. The resulting data were analyzed using the Kaluza Analysis software (Beckman Coulter, version 1.3.14026.13330), including the automatic spillover-matrix calculation feature. All samples were gated according to the strategy shown in Supplementary Figure S1. Briefly, a period with stable sample flow was chosen, and debris and doublets were removed according to the scatter signals before the dead cells were removed based on the DAPI signal. CD45 negative cells were chosen, and gates were drawn according to the shape of the population of the used FMO or unstained controls. Gates were adjusted to contain up to 0.5% false positive events. The population distribution was calculated using Boolean logic in percent of CD45 negative cells as follows: BCSPs = CD45^−^ AND ((NOT CD146^−^) AND PDPN^+^), CPs = CD45^−^ AND ((CD146^−^ AND PDPN^+^) AND ((((NOT CD73^+^) AND (NOT CD164^+^)) OR (CD73^+^ AND (NOT CD164^+^))) OR ((NOT CD73^+^) AND CD164^+^))), Ops = CD45^−^ AND ((NOT CD146^−^) AND (NOT PDPN^+^)), SSCs = CD45^−^ AND (((CD146^−^ AND CD73^+^) AND CD164^+^) AND PDPN^+^).

### 2.6. Osteogenic Differentiation

SSCs at passage 2 or 3 were seeded on well plates precoated with collagen type 1 fibers with a density of 5 × 10^3^ cells. The SSCs were fed with an osteo inductive medium (OIM) consisting of DMEM high glucose, 10% sterile filtered fetal bovine serum (FBS), 1% penicillin/streptomycin, 2 mM L-glutamine, 100 nM dexamethasone (Sigma Aldrich, St. Louis, MO, USA), 10 mM sodium β-glycerophosphate (Sigma Aldrich, St. Louis, MO, USA), and 2.5 mM ascorbic acid 2-phosphate (Sigma Aldrich, St. Louis, MO, USA) every three days for 7, 10, or 14 days.

To investigate osteogenic differentiation under proinflammatory conditions, the cells were fed with OIM supplemented with 100 ng/mL TNF-α (Peprotech, Cranbury, NJ, USA) or 10 ng/mL TNF-α or 10 ng/mL IL-1β (Peprotech, Cranbury, NJ, USA). Osteogenic potential was assessed by detecting calcium deposition, visualized with alizarin red staining (ScienCell, Carlsbad, CA, USA). For quantification, alizarin red was extracted with acetic acid followed by photometric measurement as shown by Gregory and colleagues [33].

### 2.7. RT-PCR

SSCs from three male and three female donors (passage 2 or 3) were osteogenically differentiated for 14 days as described above, followed by RNA isolation of pooled cell pellets for each male and female SSCs with the NucleoSpin RNA XS Kit (Macherey–Nagel, Dueren, Germany) according to the manufacturer’s guidelines. Quality and quantity verification was performed via NanoDrop (Thermo Fisher Scientific). Next, a first-strand cDNA synthesis kit (Thermo Fisher Scientific) was used to generate complementary DNA (cDNA). Subsequently, PCR was performed using Taq DNA polymerase with ThermoPol^®^ Buffer (New England Bioloabs, Frankfurt am Main, Germany) with the following primers for COL1A1 (CAGCCGCTTCACCTACAGC, TTTTGTATTCAATCACTGTCTTGCC), Osteopontin (AGCCAGGACTCCATTGACTCGAA, GTTTCAGCACTCTGGTCATCCAGC), Runx2 (CACTCACTACCACACCTACC, TTCCATCAGCGTCAACACC), TGF-ß1 (CAGTACAGCAAGGTCCTTGC, ACGTAGTAGACGATGGGCAG), ALPL (CTACCTGTGTGGGGTGAAGG, GGGCATCTCGTTGTCTGAGT), and GAPDH (CATGAGAAGTATGACAACAGCCT, AGTCCTTCCACGATACCAAAGT). PCR fragments were loaded on a 2% agarose gel (GENAXXON bioscience, Ulm, Germany), followed by electrophoresis for 45 min at 100 volts.

### 2.8. Statistical Analysis

All statistically analyzed data were initially tested for normal distribution. The Kruskal–Wallis test was used for non-parametric data, whereas normal distributed data were analyzed using ordinary one-way ANOVA utilizing the Graph Pad Prism software (GraphPad Software, Version 9.5.1, 2023, San Diego, CA, USA). Quantification of alizarin red S was performed with three replicates.

## 3. Results

### 3.1. Isolation of Human Skeletal Stem Cells from Femoral Heads

**Figure 1:**
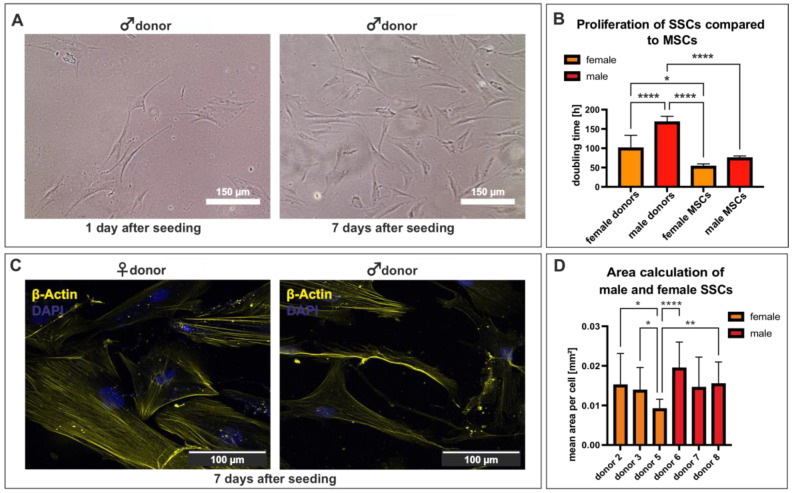
Isolated SSCs (p2-3) grew adherently with an enlarged morphology. (**A**) SSCs of both sexes grew adherently with an enlarged morphology. (**B**) Male and female SSCs revealed a slightly increased doubling time compared to MSCs of both sexes. Mean +/− SD. Ordinary one-way ANOVA, * *p* < 0.0332, **** *p* < 0.0001, was considered significant. (**C**) β-actin expression revealed enlarged morphology of male and female SSCs. (**D**) The SSCs of female donor 5 grew significantly smaller compared to the cells of donors 2, 3, 6, and 8. Mean +/− SD Kruskal–Wallis test, * *p* < 0.0332, ** *p* < 0.0021, **** *p* < 0.0001, was considered significant.

Femoral heads were obtained during arthroplasty surgery from age-matched adult donors from both sexes (Table 1). After mechanical shredding and enzymatic digestion, a density gradient was used to separate the skeletal stem cells, followed by adherent cultivation in HPL-containing medium (Figure 1). To determine the doubling time of the skeletal stem cells, we performed proliferation assays on male and female SSCs at passage 2–3. The cells revealed an enlarged adherent morphology one day after seeding, which was maintained (Figure 1A). The proliferation assays were performed with cells derived from four male and four female donors and compared to each other. As a control, human mesenchymal stem cells of both sexes were used. The male and female SSCs revealed a significantly higher doubling time compared to the MSCs of both sexes (Figure 1B). Interestingly, the male populations, with a mean doubling time of 168.0 h, grew significantly slower as compared to their female counterparts with a mean doubling time of 100.6 h (Figure 1B). To further investigate the enlarged morphology depicted in Figure 1A, we performed immunocytochemistry with an antibody against the cytoskeletal marker β-actin after 7 days of cultivation of three male and three female SSCs at passage 2–3 (Figure 1C). Next, we calculated the area of the cells as described in Section 2. Female SSC areas ranged from 0.005 to 0.032 (mean: 0.0091–0.0151) mm^2^, whereas the male SSC areas ranged from 0.01 to 0.036 (mean: 0.0145–0.0193 mm^2^) (Figure 1D). Donors 2, 3, 6, and 8 grew significantly larger than donor 5, which had the smallest cellular area of all compared donors (Figure 1D). In summary, female SSCs grow significantly faster and in a smaller phenotype compared to their male counterpart.

### 3.2. Skeletal Stem Cells Express Stem Cell Markers and Lack the Expression of Neural Crest Markers

**Figure 2:**
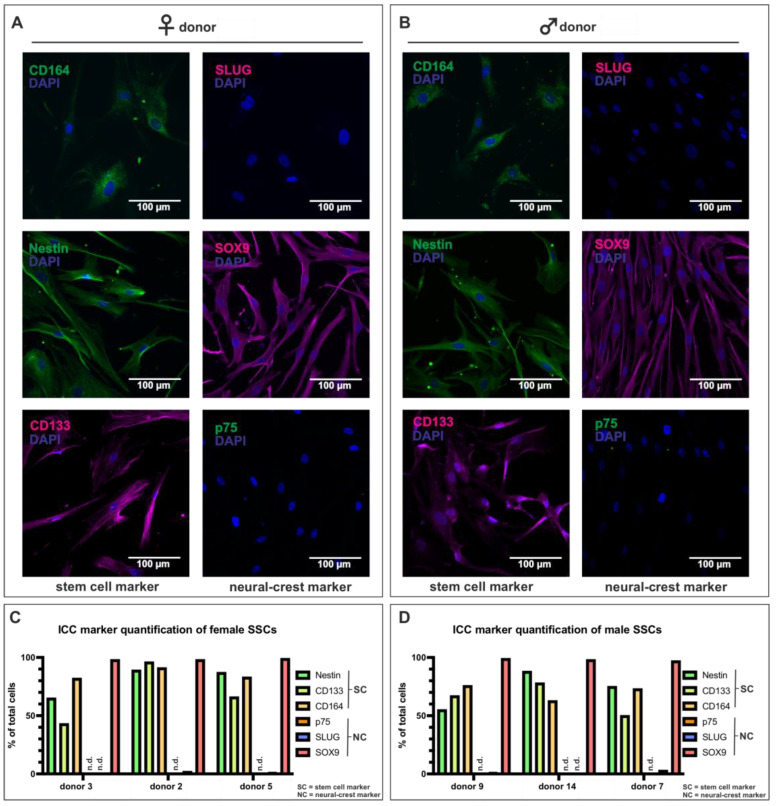
SSCs (p2-3) expressed stem cell markers and lacked neural crest markers. (**A**) SSCs derived from female donor 2 expressed CD164, Nestin, CD133, and SOX9 and lacked the expression of SLUG and p75. (**B**) Male donor 14-derived SSCs expressed CD164, Nestin, CD133, and SOX9 but did not express SLUG or p75. (**C**) Semiquantitative analysis showed high Nestin (65.6–89.4%), CD133 (43.5–96.3%), CD164 (82.7–91.0%), and SOX9 (98.1–99.3%) expression in three female SSC populations but lacking p75 (0.0–0.0%) and SLUG (1.1–2.5%) expression. (**D**) Semiquantitative analysis of three male SSC populations unraveled high levels of Nestin (55.4–88.2%), CD133 (50.9–78.8%), CD164 (62.8–75.8%), and SOX9 (97.1–99.0%), but p75 (0.0–0.0%) and SLUG (1.1–3.2%) expressions were deficient.

After successful isolation, the skeletal stem cells of both sexes (p2-3) were characterized using immunocytochemistry, regarding several stem cell and neural crest markers. The immunocytochemistry images of female SSCs showed a close to ubiquitous expression of the stem cell markers CD164, Nestin, and CD133 (Figure 2A, left side). Interestingly, of the neural crest markers, only SOX9 was expressed in female SSCs; SLUG and p75 were not (Figure 2A right side). In line with this, the immunocytochemistry images of male SSCs revealed similar results. Most of the cells expressed CD164, Nestin, CD133, and SOX9, while SLUG and p75 were only sparsely expressed (Figure 2B). Furthermore, the semiquantitative analysis of marker expression in the immunocytochemistry images revealed a high expression of Nestin (65.6–89.4%), CD133 (43.5–96.3%), CD164 (82.7–91.0%), and SOX9 (98.1–99.3%), accompanied by a lack of expression of p75 (0.0–0.0%) and SLUG (1.1–2.5%), for the female SSCs (Figure 2C). The male SSC populations corroborated these findings, demonstrating strong expression of Nestin (55.4–88.2%), CD133 (50.9–78.8%), CD164 (62.8–75.8%), and SOX9 (97.1–99.0%), as well as low expression of p75 (0.0–0.0%) and SLUG (1.1–3.2%) (Figure 2D).

### 3.3. Flowcytometric Analysis of Male and Female SSCs Revealed Heterogenous Populations of Stem Cells and Progenitors

**Figure 3:**
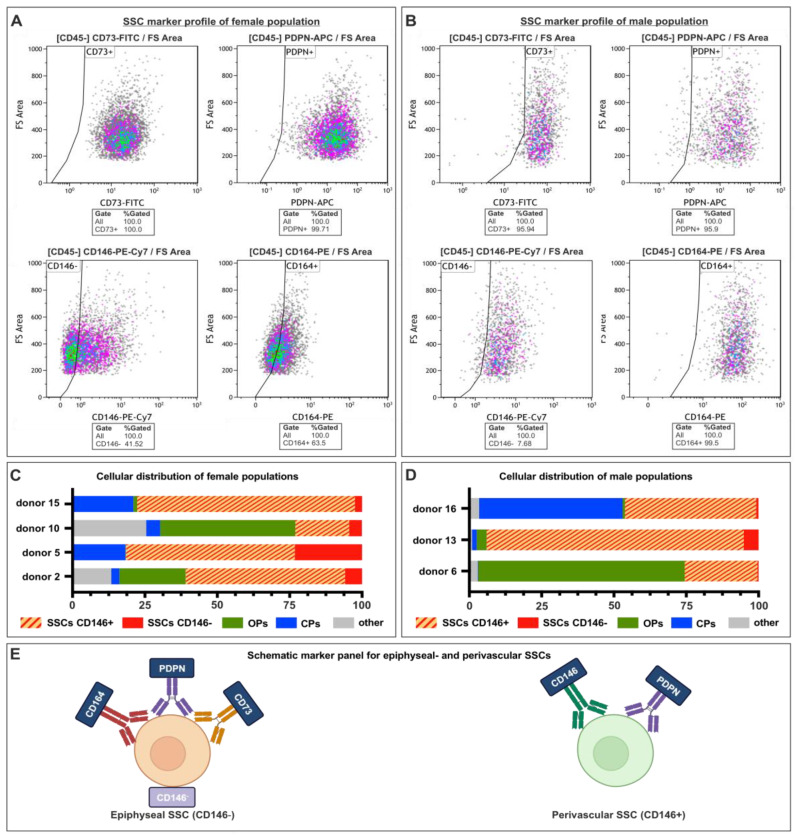
Female and male SSCs expressed a specific marker profile. (**A**) SSCs derived from female donors showed specific marker expression for skeletal stem cells. (**B**) SSCs derived from male donors demonstrated specific marker expression for skeletal stem cells. (**C**) Cells derived from female donors contain CD146^−^ SSCs, CD146+ SSCs, CPs, OPs, and other cells. (**D**) Cells derived from male donors contain CD146^−^ SSCs, CD146+ SSCs, CPs, OPs, and other cells. (**E**) Schematic depiction of marker panels for epiphyseal and perivascular SSCs.

Following the immunocytochemistry-based characterization, the SSCs derived from the male and female donors (p1) were subjected to flow cytometry analysis to determine their SSC marker profile. Initially, duplicates, debris, and dead cells were removed from the analyses. As expected, the populations lacked the hematopoietic marker CD45 (see Figure S2). For example, in the SSCs obtained from the female donor 5, we observed that 40% of the CD45 negative population exhibited a lack of CD146 expression. The vast majority of the cells expressed the markers CD73 and PDPN. Specifically, CD73 was expressed by 100% of the total cell population, while PDPN was expressed by 100% of the total cell population (Figure 3A). It is noteworthy that a significant proportion of cells, specifically 63%, exhibited positive expression of CD164, a finding that was consistent with the results obtained from the previous ICC analysis, as depicted in Figure 2C and Figure 3A. Four additional female SSC populations were examined using the marker panel previously reported. The analysis revealed that these populations, in addition to SSCs, also contained OPs, CPs, and other cells, while the majority of cells (up to 70%) are SSCs (Figure 3C). In addition, it is noticeable that the proportions of the respective cell types varied with the associated donor (Figure 3C).

Our study also covered the examination of male SSCs. The gating strategy, as depicted in Figure S2, was also applied to the SSCs obtained from male donor 13, as shown in Figure 3B. A minority of individuals within the population had a CD146 negative phenotype, accounting for approximately 88% of the total population. Upon examination of the remaining markers, it was evident that CD73 was expressed in 100% of the samples, PDPN in 96% of the samples, and CD164 in 100% of the samples (Figure 3B). In addition, a comprehensive analysis was conducted on a total of three male donors. The data revealed a conspicuous disparity in the quantities of SSCs amongst the male populations with a notable prevalence of perivascular SSCs (donor 13: 89%) or OPs (donor 6: 71%) (Figure 3D).

### 3.4. SSCs of Both Sexes Are Capable of Rapid Osteogenic Differentiation

**Figure 4:**
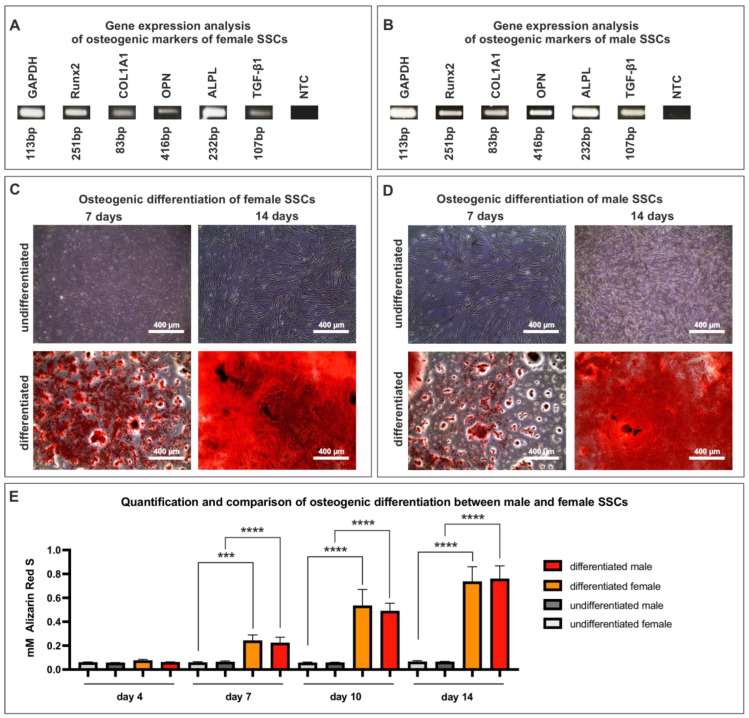
SSCs of both sexes (p2-3) have the capacity of rapid osteogenic differentiation. (**A**) Gene expression analysis of female SSCs after 14 days of osteogenic differentiation. (**B**) Gene expression analysis of male SSCs after 14 days of osteogenic differentiation. (**C**) Osteogenic differentiation of female SSCs for 7 or 14 days resulted in calcified areas (depicted in red). (**D**) SSCs obtained from male individuals underwent osteogenic differentiation for 7 or 14 days, resulting in calcified regions (depicted in red). (**E**) Quantification of three female and three male SSC populations demonstrated early osteogenesis starting at day seven without significant differences between the sexes. Mean +/− SD. Ordinary one-way ANOVA, *** *p* < 0.0021, **** *p* < 0.0001, was considered significant.

To determine the osteogenic potential of the SSCs under physiological conditions, differentiation assays were performed with male and female SSCs (p2-3). After 14 days of osteogenic differentiation, gene expression analysis was performed with osteogenic markers, such as Runx2, COLA1A, osteopontin (OPN), alkaline phosphatase—biomineralization associated (ALPL), and TGF-β1. Specific bands for all markers are visible in both male and female SSCs (Figure 4A,B). In addition to gene expression, osteogenic differentiation was verified by analyzing calcium deposition using alizarin red staining. After one week of induced osteogenic differentiation performed with female SSCs, red spots were detectable, indicating a successful calcification (Figure 4C). After two weeks, the red spots became a constant red staining, signifying full osteogenesis. (Figure 4C). Next, male SSCs showed similar results in their osteogenic potential. After one week of directed osteogenic differentiation, the male SSCs revealed red areas, indicating successful osteogenesis (Figure 4D). Matching to their female counterpart, at the end of two weeks, the red patches had turned into a continuous crimson stain, indicating complete osteogenesis (Figure 4D). Quantification of three female SSC populations revealed a significant increase in alizarin red S concentration starting at seven days of differentiation, as compared to the undifferentiated control (Figure 4E). Accordingly, the quantification of three male SSC populations was also significant starting at seven days of osteogenic differentiation, as compared to the undifferentiated control (Figure 4E). When comparing male to female SSCs in terms of osteogenic potential, no statistically significant difference could be observed (Figure 4E). Upon increased passages, we noticed a decreasing osteogenic potential.

### 3.5. Proinflammatory Cytokines Stimulate Nuclear Translocation of NF-κB in SSCs

**Figure 5:**
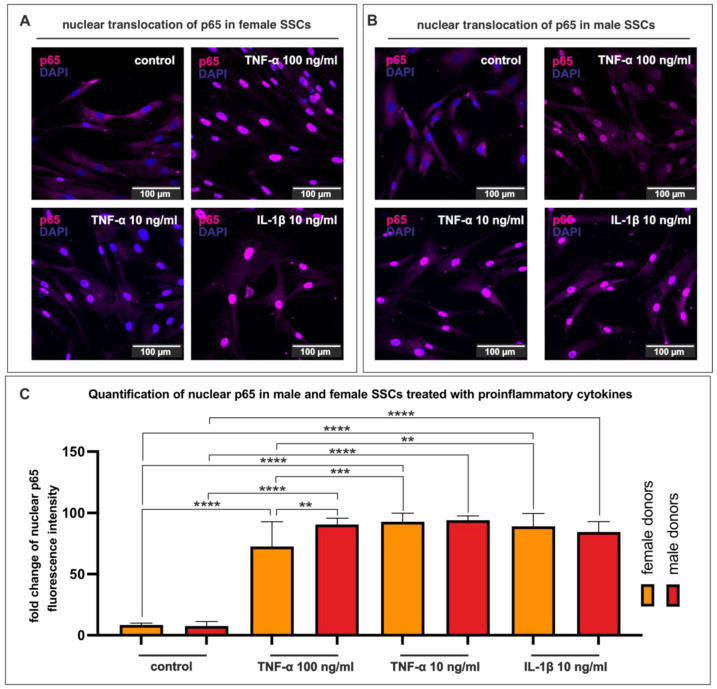
Stimulation of SSCs (p2-3) with proinflammatory cytokines results in nuclear translocation of NF-κB p65. (**A**) Immunocytochemistry of female SSCs demonstrated a nuclear translocation of p65 upon stimulation with either 100 ng/mL TNF-α or 10 ng/mL TNF-α or 10 ng/mL IL-1β for 30 min. (**B**) Upon stimulation with 100 ng/mL TNF-α or 10 ng/mL TNF-α or 10 ng/mL IL-1β for 30 min, male SSCs exhibited a nuclear translocation of p65 compared to the solvent control. (**C**) Stimulation of three male and three female SSC populations with proinflammatory cytokines resulted in a significant nuclear translocation of p65. A total of 100 ng/mL TNF-α activated nuclear p65 in male SSCs significantly higher than in their female counterparts. Ordinary one-way ANOVA, ** *p* < 0.0332, *** *p* < 0.0021, **** *p* < 0.0001, was considered significant. Mean +/− SD.

Next, we looked for sex-specific variations in SSCs during osteogenic differentiation in an inflammatory setting. First, we confirmed that proinflammatory cytokines trigger both sexes’ SSCs (p2-3). TNF-α and IL-1β, well-known proinflammatory cytokines, were used to study the nuclear translocation of the NF-κB subunit p65, a major regulator of the inflammatory response. Female SSCs showed strong nuclear fluorescence of p65 after stimulation with either 100 ng/mL TNF-α or 10 ng/mL TNF-α or 10 ng/mL IL-1β for 30 min compared to a solvent control without stimulation (Figure 5A). Immunocytochemistry of male SSCs revealed similar results in terms of nuclear translocation of NF-κB p65 after stimulation with proinflammatory cytokines for 30 min. Treatment of male SSCs with either 100 ng/mL TNF-α or 10 ng/mL TNF-α or 10 ng/mL IL-1β for 30 min revealed a strong nuclear fluorescence compared to the untreated solvent control (Figure 5B). Quantification of three male and three female SSC populations treated with proinflammatory cytokines affirmed the previous observations with statistical significance (Figure 5C). Additionally, the quantification revealed a significant difference in nuclear translocation of p65 between the two sexes under the higher concentration of TNF-α (100 ng/mL). SSCs derived from male donors showed a stronger activation of p65 upon chronic inflammation simulated with 100 ng/mL TNF-α as compared to their female counterparts (Figure 5C). Interestingly, stimulating female SSCs with the lower dose of TNF-α (10 ng/mL) or with 10 ng/mL IL-1β resulted in a significantly increased amount of nuclear p65 fluorescence signal when compared to the higher concentration (100 ng/mL) (Figure 5C).

### 3.6. Inflammation Does Not Affect the Osteogenic Potential of SSCs in a Sex-Dependent Manner

**Figure 6:**
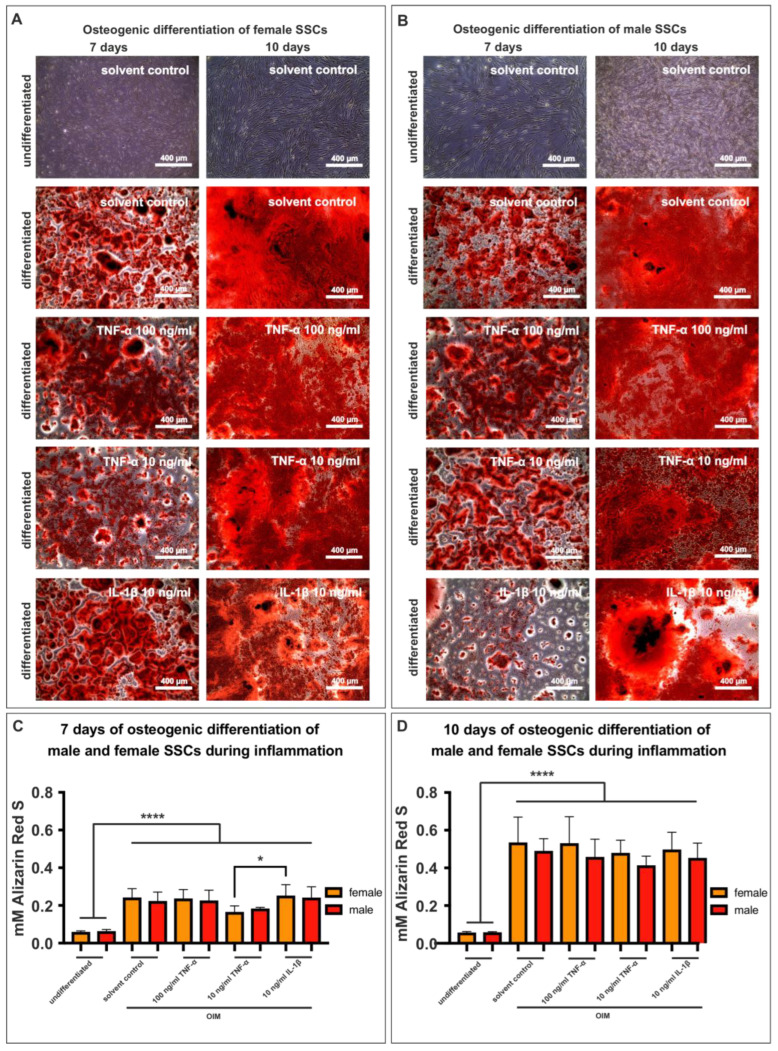
Proinflammatory cytokines did not affect SSCs (p2-3) in a sex-dependent manner. (**A**) Red dots indicated successful osteogenic differentiation of female SSCs after seven days. (**B**) Male SSCs demonstrated positive osteogenic differentiation. (**C**) The osteogenic differentiation of three female and three male SSC populations was quantified over a period of 7 days. The results indicated a reduction in alizarin red concentration following the treatment with 10 ng/mL TNF-α in the female cells only. Ordinary one-way ANOVA, * *p* < 0.05, **** *p* < 0.0001, was considered significant. Mean +/− SD. (**D**) After 10 days of osteogenic differentiation, no significant difference in terms of osteogenic differentiation between the sexes was observable, regardless of the addition of proinflammatory cytokines. Ordinary one-way ANOVA, **** *p* < 0.0001, was considered significant. Mean +/− SD.

Following the successful examination of the responsiveness of SSCs to inflammatory cytokines through NF-κB signaling, we proceeded to explore the impact of an inflammatory milieu on the osteogenic capacity of both male and female SSCs (p2-3). Consequently, we added TNF-α (at concentrations of 100 ng/mL or 10 ng/mL) or IL-1β (10 ng/mL) into the culture medium every 2–3 days. After a period of seven days, the differentiated culture of female SSCs exhibited the presence of red dots, which can be interpreted as a positive indication of effective osteogenic differentiation. Strong red staining was observed in the conditions supplemented with proinflammatory cytokines (Figure 6A, left panel). A subtle decrease in red staining was observed in the experimental group treated with a concentration of 10 ng/mL of TNF-α, as depicted in Figure 6A (left panel). Following a 10-day period of osteogenic differentiation, a significant augmentation in red staining was observed in all approaches except for the undifferentiated control (Figure 6A, right panel). Subsequently, an examination of identical conditions was conducted on male SSCs. Like their female counterparts, the male SSCs exhibited red patches in both the differentiated group and the groups that underwent treatment with proinflammatory cytokines (Figure 6B). Afterwards, the osteogenic differentiation of three male and three female SSC populations was quantified. In accordance with the findings above, it could be observed that, after 7 days of osteogenic differentiation in female SSCs, the lower dose of TNF-α (10 ng/mL) resulted in less calcium deposition compared to the treatment with IL-1β (10 ng/mL) (Figure 6C). When considering the comparison of male and female SSC populations after 10 days of osteogenic differentiation, it becomes apparent that SSCs of both sexes showed no significant disparities in an inflammatory environment (Figure 6D).

## 4. Discussion

This study focused on the examination of sex-specific differences in the cellular characteristics and osteogenic potential of adult human skeletal stem cells. First, we described the isolation of SSCs in which potent cell populations were obtained after density gradient centrifugation, followed by cultivation with HPL-supplemented medium and further characterization. We tested the hypothesis of inherent disparities in morphology, growth, or marker expression between the sexes. We found significant sex-specific variations in the doubling times among the sexes and individuals. The SSCs derived from male donors demonstrated a significantly higher doubling time (168.0 h) than that in the SSCs derived from female donors (100.6 h). It is noteworthy that a study by Lysdahl and coworkers revealed that the pH indicator phenol red (PR), which we utilized in our culture medium, known for its estrogen-mimetic properties, has no effect on the cellular viability and phenotype of male MSCs [34]. However, the exact effect of phenol red in MSC and SSC cell culture is still a matter of debate and should be addressed in future research. Focusing on sex-specific differences, Siegel and colleagues have demonstrated that female bone marrow-derived mesenchymal stromal cells (BM-MSCs) had a higher rate of growth (96 h) compared to that of their male counterparts (up to 216 h) [35]. Furthermore, female populations had a higher proportion of small, faster-proliferating cells compared to male populations, thus corroborating our findings [35].

Besides the sex, the effects of donor age on stem cell behavior and characteristics are still a matter of debate. Fickert and coworkers reported that the proliferation of mesenchymal stem cells ex vivo is not influenced by age [36]. In contrast, Zaim and colleagues demonstrated a decreasing efficiency of differentiation capacity and proliferation of human MSCs linked to increasing donor age [37]. To exclude potential age-dependent effects in our study, we specifically investigated potential sex-specific differences among age-matched donors.

For detailed characterization, we analyzed various stem cell and neural crest markers with immunocytochemistry. First, we investigated the expression of CD133 and Nestin, which are already used as established stem cell markers for various human tissues [38,39]. Our data revealed an observable variation in CD133 and Nestin expression based on the donor. Accordingly, the presence of CD133 expression in mesenchymal stem cells (MSCs) derived from both bone marrow and umbilical cord blood was found to be correlated with their capacity for osteoblast development [40]. Furthermore, the work performed by Xi and coworkers demonstrated that Nestin-positive mesenchymal stem cells (MSCs) play a role in endochondral ossification and persist as quiescent cells inside the bone marrow, while still maintaining the ability to differentiate into three lineages [39]. The high expression levels of Nestin in our investigated SSCs may contribute to potential regenerative processes of the skeleton. Since Nestin is also an established neural crest marker, we further examined the cell populations for their potential neural crest origin [41]. In addition to Nestin, we examined SOX9, SLUG, and p75 expression, which were linked to the characterization of neural crest-derived stem cells [42,43]. While SLUG and p75 were not found in the donor populations, excluding an association with the neural crest, SOX9 could be detected ubiquitously. This expression of SOX9 is of major importance as it has a crucial impact on the potential for osteogenic differentiation. This is primarily caused by the ability of SOX9 to activate RUNX2, which serves as the master transcription factor regulating osteogenic development and growth [44,45]. We additionally investigated the expression of CD164, a crucial marker utilized in the characterization of skeletal stem cells [8]. The majority of cells examined in our study showed expression of CD164, confirming their SSC origin.

To confirm our immunocytochemistry findings, we used flow cytometry to analyze the marker panel for skeletal stem cells (CD146^−^, CD73^+^, PDPN^+^, CD164^+^), as established by Chan and colleagues, in our cell populations [8]. It is notable that podoplanin (PDPN) and CD73 were expressed for up to 100% in both female and male cell populations. Upon examining the expression of CD146 and CD164, a donor-specific difference was observable. The expression of CD146 can be attributed to variability in the niche of the skeletal stem cells. CD146^−^ SSCs are mostly located in the niche of the epiphyseal region, whereas CD146^+^ SSCs are predominantly found in the perivascular areas. The presence of CD146^+^ populations can be explained by the presence of perivascular skeletal stem cells from the metaphysis during the isolation process [4,46,47]. Ambrosi and colleagues also described that the CD146^−^ SSCs may give rise to CD146^+^ multipotent skeletal progenitors, explaining our high numbers of CD146^+^ SSCs [4]. Moreover, the correlation between the flow cytometric measurement of the skeletal marker CD164 and the findings from our immunocytochemistry analysis affirmed our previous results.

After successful immunological characterization, we analyzed a potential sex dimorphism in the osteogenic differential potential of female and male SSC populations under physiological conditions. At first, we analyzed gene transcription after 14 days of osteogenic differentiation in male and female SSCs. We observed expression of RUNX2, COL1A1, OPN, ALPL, and TGF-β1 in SSCs derived from both sexes. RUNX2 is a key regulator during osteogenesis that is essential for the maturation of osteoblasts and transcription of COL1A1, which is responsible for matrix mineralization, in SSCs of both sexes [48]. ALPL is known to be involved in the differentiation process of MSCs towards osteoblasts, accompanied by regulating matrix mineralization [49]. In line with our findings, intracellular OPN expression is linked to cell migration and activation of MAPK signaling, activating RUNX2 signaling [50]. Finally, high expression of TGF-β1 promotes bone mineralization and involvement in calcium homeostasis [51]. Taken together, we observed no sex-specific difference in the gene expression patterns of osteogenic markers during osteogenic differentiation.

Additionally, strong and fast ossifications were detectable in both male and female SSCs after only seven days of directed osteogenic differentiation.

In contrast, a sexual dimorphism in the human skeleton and its homeostasis is well documented [24,52]. In general, women achieve a lower peak bone mass compared to that in their male counterparts, resulting in a two- to four-fold greater incidence of fracture and osteoporosis [53,54]. In particular, females have a higher number of osteoclasts accompanied by an accelerated osteoclast differentiation in vitro [55]. The reasons for these differences in bone mass and metabolic diseases are multitude, including the interaction of genetics, age, environment, cellular behavior, and sex hormones [56]. Several studies demonstrated that estrogen is a potential factor that can influence the osteogenic differentiation of stem cells [57,58,59,60]. In particular, Hong and coworkers demonstrated that supplementation of male and female MSCs during osteogenic differentiation with 17-β estradiol resulted in an upregulated expression of osteocalcin and ALP accompanied by increased calcium deposition [60]. Moreover, Andrew and colleagues were able to demonstrate that supplementation with estradiol in postmenopausal SSCs led to a significant increase in osteogenic differentiation [21]. Nevertheless, we only used phenol red in our differentiation culture to analyze cell autonomous effects only, which had no effect on osteogenic differentiation of SSCs of both sexes, which may be explained by a low binding affinity of phenol red to the estrogen receptor (0.001% of estradiol) demonstrated in human breast cancer cells [61]. Accordingly, Lee and coworkers reported that there were no sex-specific differences in the osteogenic differentiation potential in human bone marrow-derived MSCs [62]. These results indicate that, although there is a clinically relevant sex-specific difference between male and female bone regeneration, the exact factors modulating this sexual dimorphism are still a matter of debate and need to be part of further investigations.

Comparing the osteogenic differentiation capacity of SSCs with other stem cell types revealed that mesenchymal stem cells require a significantly longer duration to achieve an equivalent level of osteogenesis. In the study conducted by Wang and coworkers, it was revealed that human bone marrow-derived mesenchymal stem cells underwent osteogenic differentiation with ossification being evident only after a period of at least 14 days [63]. Accordingly, Hauser and colleagues tested the capacity of neural crest-derived stem cells (ITSCs) and revealed alizarin red depositions only after 18 days of directed osteogenic differentiation [43]. Thus, the osteogenic potential of SSCs is extremely fast and potent compared to MSCs and ITSCs, which have the slowest potential in this regard. In addition to cell autonomic effects that could affect the different efficiency of osteogenic differentiation, the composition of the differentiating medium could also play a key role. Some studies have shown that the replacement of FCS with HPL may have a positive effect on osteogenic differentiation. In a study conducted by Karadjian and colleagues, it was shown that there was an increased ALP activity and calcification in human MSCs due to the exchange of FCS with HPL in the differentiation medium [64]. Moreover, Mollentze and coworkers discussed in their review that the utilization of xeno-free differentiation modulators, such as HPL, should be considered in in vivo studies with prospects for later clinical applications [65].

One possible explanation for the accelerated cell intrinsic differentiation of SSCs in comparison to MSCs or ITSCs is their ability to undergo direct differentiation into bones by intramembrane ossification in addition to the conventional endochondral ossification pathway, including cartilage-to-bone transformation. In this context, a murine study reported the existence of a distinct stem cell population originating from the periosteum, known as periosteal stem cells (PSCs), which play a crucial role in the process of bone formation by direct intramembranous ossification [66] Furthermore, a significant proportion of undifferentiated precursor cells, which are present in the population as shown by flow cytometry, supports a faster osteogenesis compared to undifferentiated stem cells like MSCs or ITSCs.

To investigate whether the osteogenic potential of SSCs varies under inflammatory conditions in a sex-specific manner, we used proinflammatory cytokines (TNF-α and IL1-β) to mimic inflammatory conditions in culture. For this, we used the signal transduction pathway of the transcription factor NF-κB, which is not only involved in processes such as proliferation, differentiation, and neuroprotection but also plays a crucial role in modulating the immune response [29,67]. We were able to demonstrate that the addition of TNF-α or IL-1β leads to a strong nuclear translocation of the NF-κB subunit p65 and, thus, stimulates an inflammatory response. In line with our findings, a previous study reported that TNF-α stimulates nuclear translocation of p65 in human cancer cells [68,69]. Accordingly, IL-1β is linked to a rapid nuclear accumulation of p65 in human hepatoma cells [70]. Multiple studies have already demonstrated the influence of sex on the immunological response. It can be generally stated that sex hormones, such as testosterone and estrogens, have an impact on the immune response. Exemplarily, testosterone provides an inhibitory influence on the immune system, whilst estrogen exerts a stimulating effect. Moreover, it has been observed that women exhibit a better antibody response [71]. When analyzing genetics, it has been observed that the X chromosome has a role in the expression of genes, such as Toll-like receptors and genes that are involved in the regulation of T and B cells [72]. In addition, the presence of 17β-estradiol could induce the secretion of TNF-α and activate NF-κB in a dose-dependent manner [73].

Having demonstrated the ability of pro-inflammatory stimulation to induce inflammation, indicated by NF-κB p65 nuclear translocation in the SSCs of both males and females, the next step involved investigation of osteogenic differentiation within this inflammatory environment. During early osteogenesis after seven days, we were able to observe that TNF-α did not increase the osteogenic potential at any concentrations. Interestingly, 10 ng/mL TNF-α leads to a slightly reduced osteogenic differentiation after seven days. Accordingly, Mo and colleagues reported a dose-dependent effect of TNF-α and IL-1β on the osteogenic differentiation of MSCs [74]. In the context of prolonged osteogenesis of the SSCs, no significant disparity based on sex was observed. SSCs derived from male and female donors exhibited notably increased levels of mineralization compared to the seven-day differentiation period. Nonetheless, the scientific community extensively investigates disparities in osteogenic differentiation between males and females due to variations in the prevalence and severity of bone-related conditions, such as osteoporosis, fracture healing disorders, and osteoarthritis, among individuals of different sexes [75,76]. In a study conducted by Møller and colleagues, a more aggressive phenotype of osteoclasts in females after menopause was observed, which may contribute to a faster development of osteoporosis [77]. Nevertheless, Leskelä and colleagues showed that increasing age in human adulthood does not affect the osteogenic potential of MSCs [78]. Here, we could extend these studies to SSCs. Our in vitro model of skeletal stem cells demonstrated that both male and female SSCs are capable of fast osteogenic differentiation, allowing for the investigation of osteogenesis without the influence of bone-absorbing osteoclasts.

## 5. Conclusions

The aim of this study was the investigation of potential sex-specific effects in human SSCs. Here, we observed significantly faster proliferation and smaller cell morphology of female SSCs compared to their male counterparts. Moreover, immunophenotyping of SSCs using immunocytochemistry revealed high expression in both sexes of CD164, Nestin, CD133, and SOX9 and the absence of the neural crest markers SLUG and p75. Flow cytometric analysis demonstrated that our populations contain mainly epiphyseal and perivascular SSCs beside some unipotent progenitors (CPs and OPs) with no differences regarding sex. We tested the SSCs for their potency in osteogenic differentiation and demonstrated strong osteogenic marker expression in both sexes. Moreover, male and female SSCs exhibited strong mineralization starting at day seven. We established an inflammatory model for the SSCs using TNF-α and IL-1β, which resulted in nuclear translocation of the NF-kB subunit p65 in both sexes. Based on this model, we determined the osteogenic differentiation potential of male and female SSCs and revealed that inflammation has no effect on osteogenic differentiation, independent of sex, in vitro. Taken together, we conclude that both sexes can regenerate bone to the same amount in vitro, independent of inflammation. This well-established in vitro stem cell model might be suitable for a sex-specific drug screening regarding the treatment of bone diseases.

## Figures and Tables

**Table 1:** Donor ID, sex, age, and diagnosis, as classified according to the Kellgren and Lawrence classification system, for the selected SSC donors.

Donor ID	Sex	Age [Y]	Diagnosis	Donor ID	Sex	Age [Y]	Diagnosis
Donor 1	female	72	coxarthrosis III°	Donor 6	male	61	coxarthrosis III°
Donor 2	female	57	coxarthrosis IV°	Donor 7	male	61	coxarthrosis III°
Donor 3	female	63	coxarthrosis IV°	Donor 8	male	59	coxarthrosis III°
Donor 4	female	57	coxarthrosis III°	Donor 9	male	62	coxarthrosis III°
Donor 5	female	67	coxarthrosis III°	Donor 12	male	40	coxarthrosis III°
Donor 10	female	68	coxarthrosis III°	Donor 13	male	62	coxarthrosis III°
Donor 11	female	63	coxarthrosis III°	Donor 14	male	54	coxarthrosis III°
Donor 15	female	60	coxarthrosis III°	Donor 16	male	62	coxarthrosis III°

## Data Availability

The data presented in this study are available upon request from the corresponding author. The data are not publicly available due to ethical and privacy reasons.

## Supplementary Materials

The following supporting information can be downloaded at: https://www.mdpi.com/article/10.3390/cells12232683/s1, Figure S1: Schematic Isolation of SSCs derived from the epiphyseal growth plate of human femoral heads after arthroplasty surgery; Figure S2: Gating strategy for SSCs multiparameter flow cytometry.
