# Supplementary material for: Analyzing Sex-Specific Dimorphism in Human Skeletal Stem Cells
Source: Cells. 2023 Nov 22;12(23):2683. doi: 10.3390/cells12232683 (PMC10705359; doi:10.3390/cells12232683)
Supplement: Supplementary file 1 [file cells-12-02683-s001.zip › Supplementary figures.pdf]

Supplementary figures:

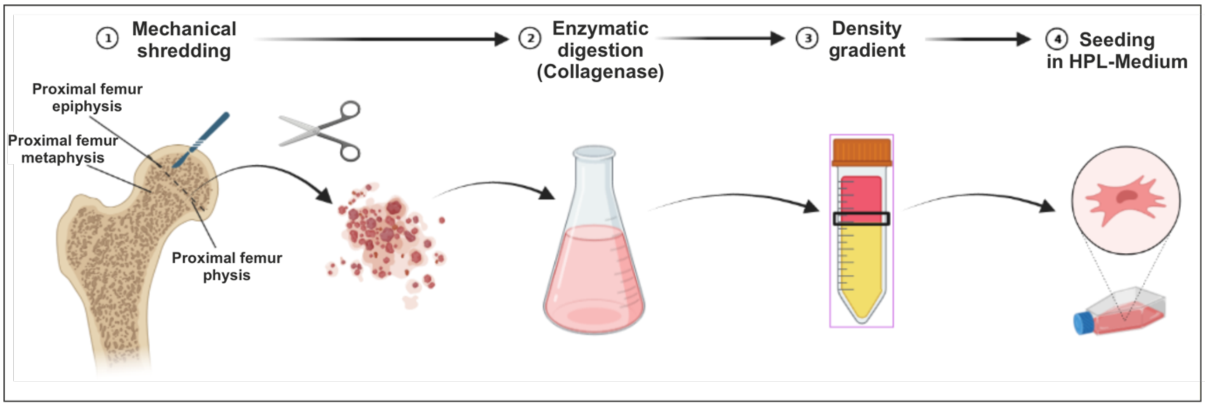


Figure S1 Schematic Isolation of SSCs derived from the epiphyseal growth plate of human femoral heads after arthroplasty surgery.

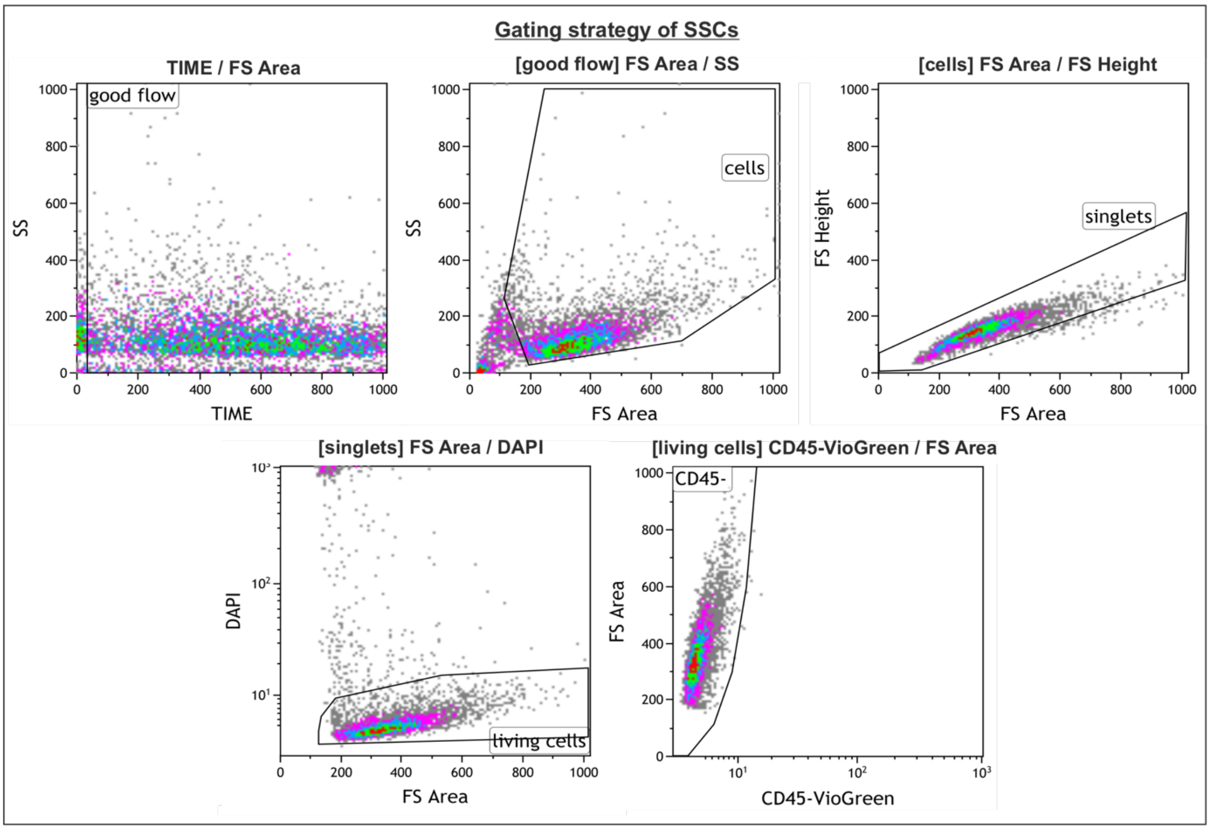


Figure S2 Gating strategy for SSCs multiparameter flow cytometry. At first, the good flow was determined by excluding unevenly distributed events. Next, debris was removed by the cells gate, followed by the determination of single cells with the singlets gate. Next, dead cells were excluded using the living cells gate containing cells without DAPI signal. Finally, CD45 positive cells were excluded using the CD45- gate.
