# Supplementary material for: Analyzing Sex-Specific Dimorphism in Human Skeletal Stem Cells
Source: Cells. 2023 Nov 22;12(23):2683. doi: 10.3390/cells12232683 (PMC10705359; doi:10.3390/cells12232683)
Supplement: Supplementary file 1 [file cells-12-02683-s001.zip › Supplementary material.pdf]

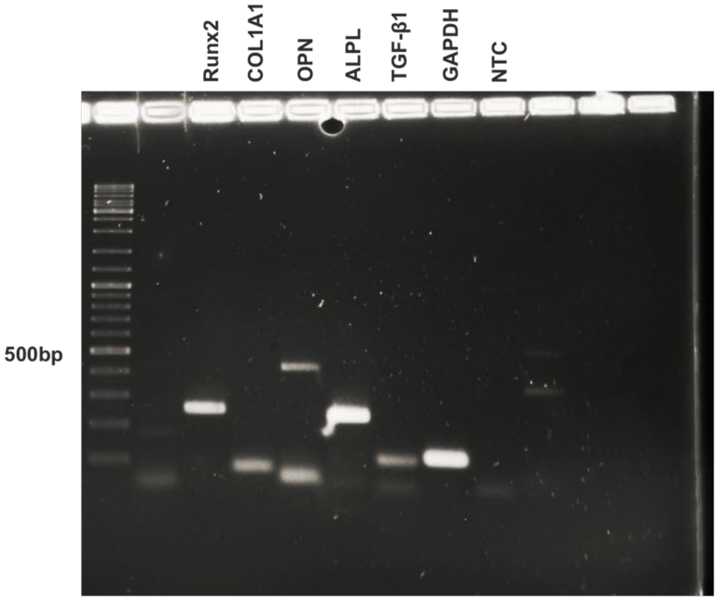

Gelblot: 14 days osteogenic differentiation of female SSCs (pooled n=3)

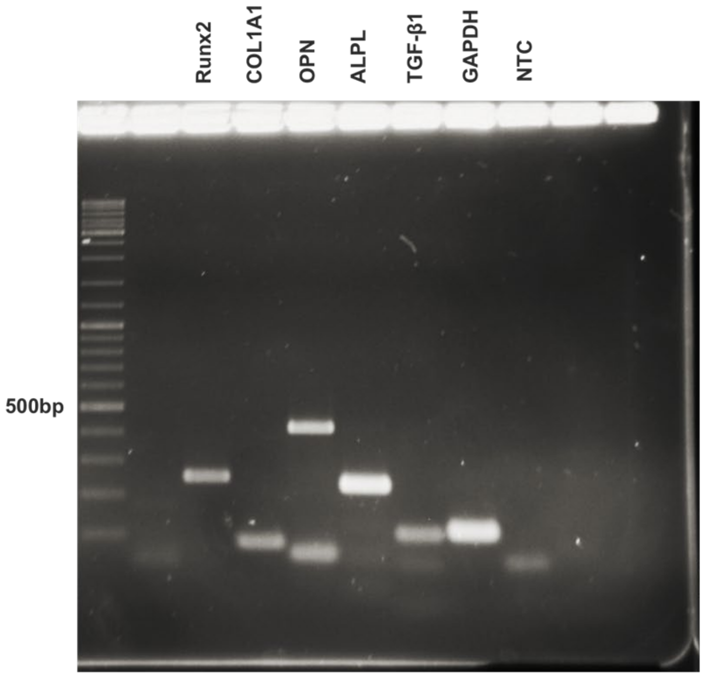

Gelblot: 14 days osteogenic differentiation of male SSCs (pooled n=3)
